# Pomegranate variety and pomegranate plant part, relevance from bioactive point of view: a review

**DOI:** 10.1186/s40643-020-00351-5

**Published:** 2021-01-03

**Authors:** Pablo Melgarejo-Sánchez, Dámaris Núñez-Gómez, Juan J. Martínez-Nicolás, Francisca Hernández, Pilar Legua, Pablo Melgarejo

**Affiliations:** Plant Production and Microbiology Department, Orihuela Polytechnical High School (EPSO), Miguel Hernandez University, Ctra. Beniel Km 3.2, 03312 Orihuela, Spain

**Keywords:** *Punica granatum* L., Bioactive compounds, Pomegranate varieties, Pomegranate parts

## Abstract

Pomegranate (*Punica granatum* L.) belongs to the Punicaceae plant family. It is an important fruit due to its nutritional and medicinal properties. Pomegranates are widely distributed around the world and, therefore, have a broad genetic diversity, resulting in differences in their phytochemical composition. The scientific community has focused on the positive health effects of pomegranate as a whole, but the different varieties have rarely been compared according to their bioactive compounds and bioactivity. This review aims to provide a holistic overview of the current knowledge on the bioactivity of pomegranate trees, with an emphasis on differentiating both the varieties and the different plant parts. This review intends to provide a general and organized overview of the accumulated knowledge on pomegranates, the identification of the most bioactive varieties, their potential consumption pathways and seeks to provide knowledge on the present gaps to guide future research. 
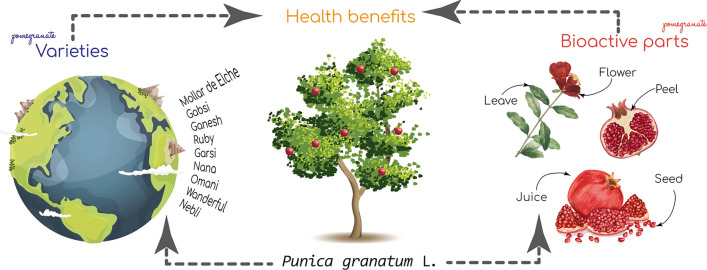

## Highlights

- Pomegranate varieties differ in phytochemical composition and bioactive capacity
- In most studies, the variety was unspecified, making its replicability difficult
- Wonderful was the variety with the highest number of bioactivities in last years
- Peel was the part that presents the most substantial amount of bioactive compounds
- It is necessary that future studies specify the varieties and parts used.

## Introduction

Pomegranates (*Punica granatum* L.) originated in the Middle East, and their cultivation was extended to different regions in the world through the propagation of its seeds, which resulted in a broad genetic diversity. The largest pomegranate germplasm collection is presently found at the Garrygala Research Station in Turkmenistan with 1117 accessions, followed by India with 810, Russia with 800, Iran with 770, Ukraine and Turkey with 370, China with 289, the USA with about 200, and Israel with 150 accessions (Still 2006; Holland and Bar-Ya’akov 2018). In the European Union, the largest germplasm collection is located in Spain, with more than 140 accessions (Zuriaga et al. 2017). New accessions have been characterized in the last few years, demonstrating the wide diversity and the growing interest on this fruit around the world (Zarei 2017; Khadivi et al. 2018). The total area of pomegranate production worldwide is estimated to be well above 300,000 ha, with more than 76% found in 5 countries (India, Iran, China, Turkey, and the USA) (Melgarejo et al. 2015). Iran, India, and China are responsible for 80% of the global production, estimated to be about 3 million tons (Ambigaipalan et al. 2016; IVIA 2017).

The primary use of pomegranate is fresh consumption; however, in the last decades, there has been an increase in demand for industrially-processed products such as juices, alcoholic drinks, jams, dehydrated seeds, nutritional fiber, dry rind for making infusions, and extracts from its different parts (Hmid et al. 2017). This growing interest in the use of pomegranate and its parts is motivated both the increasing demand by the nutrition, pharmaceutical, and cosmetic industries (Karimi et al. 2017) in addition with the incipient interest of consumers for its fresh consumption. During 2018, the consumption of fresh fruit in Spain accounted for 9.3% of the average budget of a household devoted to food (MAPA 2019).

This increasing importance may be due to the latest scientific research studies, which have indicated that pomegranates contain substances with antimicrobial, anti-cancer, cardio-protective, and anti-inflammatory activity. Also, the plant could be used for the treatment of diabetes mellitus and obesity, and can also improve sperm quality, among other uses (Govindappa 2015; Hosseini et al. 2016; El-Sheshtawy et al. 2016; Ghavipour et al. 2017; Gbinigie et al. 2017; Khwairakpam et al. 2018; Lepionka et al. 2019; Mohamad Sukri et al. 2019).

The pomegranate fruit is considered to be part of the so-called *Super Fruits* group, which is a term used to highlight the excellent nutritional qualities and health-promoting phytochemicals of certain fruits (Fischer et al. 2011; Czieczor et al. 2018; Kumar and Neeraj 2018). This is perhaps the reason why pomegranates have been used for pharmaceutical purposes, since ancient times, and it continues today, as pharmaceutical companies are extracting the bioactive compounds of the fruit to create capsules for dietary supplementation (Sidhu and Zafar 2012; Karimi et al. 2017).

Pomegranates contain many bioactive compounds such as alkaloids, ellagic acid, punicalagin among other ellagitannins, anthocyanins, flavonoids, tannins, and other phytochemicals that may play an essential role in human health and the prevention and treatment of many illnesses (Setiadhi and Sufiawati 2017). Different varieties normally have different physico-chemical characteristics and may, therefore, differ in the amount and types of bioactive compounds (Li et al. 2015; Hmid et al. 2017). Thus, the bioactive profile is influenced by the cultivar, growing region, climate, maturity, cultivation practice, and storage conditions (Fernandes et al. 2017).

The aim of this review is to analyze and summarize the recent research studies conducted with pomegranates to identify the main bioactive compounds present in the different varieties and plant parts (fruits, flowers and leaves). This will allow us to elucidate the consumption trends and/or preferences in relation with both the pomegranate variety and pomegranate part consumed (that is related with the type of consumption) to obtain health benefits.

## Evolution of scientific interest

A brief systematic literature search was carried out which aimed to identify the evolution of pomegranate scientific research, its applications, areas of interest, characteristics, and pomegranate varieties. The study was performed through the analysis of results from the online databases Scopus and Web of Science, in April, 2019, using “*Punica granatum*” AND “pomegranate” as the main search keywords, although the complete term “*Punica granatum* L.” was also utilized. This study followed the PRISMA guidelines for systematic reviews (Moher et al. 2015).

The search results showed a considerable increase in scientific studies focused on pomegranate. Thus, since 1967, more than 6000 works have been published. The most remarkable increase was in the last 20 years (> 90%), indicating the relevance and current nature of the subject. Relevant differences were observed depending on the database consulted (WoS > Scopus) and the keywords analyzed (pomegranate > *Punica granatum* > *Punica granatum* L.). The most significant number of articles were for the generic word "pomegranate" (7143 papers in WoS) when compared to "*Punica granatum* L." (1362 papers in WoS). This difference may be motivated by the aims of the publications, as most of them perhaps only focused on a specific part, function, and/or characteristic and not on the plant itself, hence the use of its generic nomenclature. Regarding the part of the plant, even though all the parts were represented, "fruit" (36%) was the most repeated term in the articles, followed by "juice" (23%), "peel" (13%), "seed" (11%), "aril" (6%), "leaves" (5%), "flower" (3%), "rind" (3%), and "pericarp" (1%).

In addition, 11,921 patents related to pomegranate and its compounds were identified, which indicate its potential for use and applications in the pharmaceutical, nutritional, medical, cosmetic, and/or industrial sectors. Therefore, general reviews such as the present one are necessary for systematizing the accumulated knowledge and for indicating further research gaps.

### Bioactive compounds

Bioactive compounds in food may be defined as phytochemicals that have an impact on metabolic processes and that may result in health benefits (International Food Information Service 2009). When assessing the health benefits of pomegranate, it is important to carry out chemical analysis that aims to identify the compounds and to elucidate whether one or several compounds, together or alone, are responsible for the effects. This is possible when specific experiments that isolate compounds are carried out, but it should also be considered that the synergistic actions of different constituents may be greater than those of single compounds (Olapour and Najafzadeh 2010).

The main bioactive compounds identified in pomegranate in the last decades and attributed, directly or indirectly, to health benefits, are summarized in Table 1, which also shows in which part of the pomegranate fruit (peel, juice, seed and rind) and tree (leaves and flowers) the compound was found and the specific bioactivities ascribed to them. It should be noted that among the studies specified in Table 1, 91% are pre-clinical studies (51% using cell models and 40% using animals) and only 9% are studies in humans. This result faithfully identifies the general trend of accumulated knowledge about bioactive compounds in pomegranate, where the laboratory knowledge is bigger than its real apply in humans health.

**Table 1:** Summary of the main bioactive pomegranate compounds found in the pomegranate peel (PP), juice (PJ), seed (PS), flower (PF), leaves (PL) and not specified part (NS) and the references, where they were found and/or studied

	Alzheimer’s disease	Anti-allergic	Antianxiety	Anticonvulsant	Anti-diabetic	Anti-inflammatory	Antimicrobial	Antinonciceptive	Anti-tyrosinase	Breast cancer	Colon cancer	Hepatocarcinogenesis	Prostate cancer	Lung cancer	References
Phenolic compounds															
Protocatechuic acid^PJ, NS^												X			(Poyrazoğlu et al. 2002; Bishayee et al. 2011)
Caffeic acid^PJ, NS^						X						X			(Bishayee et al. 2011; AlMatar et al. 2019)
Ferrulic acid^PP, NS^						X						X			(Bishayee et al. 2011; Wang et al. 2014; Hmid et al. 2018)
Trans-p-coumaric acid ^NS^												X			(Bishayee et al. 2011)
Unspecified phenols ^PP^														X	(Jayakumar et al. 2012)
Unspecified Tannins^PP, PL^			X	X		X	X	X							(Das and Barman 2012; Ouachrif et al. 2012; Fawole et al. 2012)
Hydrolyzable tannins & derivatives															
Unspecified^NS^					X	X	X		X						(Kutan Fenercioglu et al. 2010; Larrosa et al. 2010)
Corilagin^NS^						X			X						(Bishayee et al. 2011; Khwairakpam et al. 2018; Zehra et al. 2019)
Gallagyldilactone ^PJ^	X	X			X		X		X	X	X				(Kasimsetty et al. 2010; Yuniarti et al. 2018)
Gallagic acid^PJ, PP^				X		X	X	X			X				(Kasimsetty et al. 2010; Heena et al. 2018)
Punicalagins (α and β)^PP, PR, NS^		X	X				X	X		X		X	X		(Rosas-Burgos et al. 2017; Suman and Bhatnagar 2019)
Punicalins^PJ^	_				X		X			X	X		X		(Kasimsetty et al. 2010; Giamogante et al. 2018)
Gallic acid^PP, PJ, NS^					X	X	X		X		X	X			(Kasimsetty et al. 2010; Arun and Singh 2012; Fawole et al. 2012; Bishayee et al. 2013; Wang et al. 2014; Banihani et al. 2014; Dludla et al. 2018)
Ellagic acid^PP, PJ, PS, NS^	X	X			X	X	X		X	X	X	X	X		(Makino-Wakagi et al. 2012; Wang et al. 2012, 2014; Fawole et al. 2012; Bishayee et al. 2013; Rojanathammanee et al. 2013; Banihani et al. 2014; Tamamm et al. 2018; Yuniarti et al. 2018)
Flavonoids															
Unspecified^PP, PL^			X	X	X	X	X	X				X			(Kutan Fenercioglu et al. 2010; Das and Barman 2012; Ouachrif et al. 2012; Fawole et al. 2012)
Catechin^PP^							X		X						(Fawole et al. 2012; AlMatar et al. 2019)
Epicatechin^PP^						X	X		X						(Wang et al. 2012; Fawole et al. 2012)
Kaempferol^PP^						X									(Wang et al. 2012)
Luteolin ^PJ^						X	X			X			X		(Lopez-Lazaro 2009; Wang et al. 2012; Rocha et al. 2012)
Quertecin ^PP^						X									(Wang et al. 2012)
Rutin ^PP^							X		X						(Fawole et al. 2012; Gullon et al. 2016)
Cyanidin 3, 5-diglucoside ^PP^							X		X						(Fawole et al. 2012)
Delphinidin 3,5-diglucoside ^PP^							X		X						(Fawole et al. 2012)
Fatty acids															
Octadecatrienoic acids ^PS^						X						X			(Bishayee et al. 2011; Coursodon-Boyiddle et al. 2012)
Punicic acid ^PJ, P S^					X	X				X			X		(Vroegrijk et al. 2011; Coursodon-Boyiddle et al. 2012; Wang et al. 2012; Rocha et al. 2012; Nekooeian et al. 2014; Banihani et al. 2014; Mphahlele et al. 2017)
Other compounds															
17-α-estradiol ^PS^												X			(Bishayee et al. 2011)
5-hydroxymethylfurfural ^NS^												X			(Bishayee et al. 2011)
Alkaloids ^PP^						X		X							(Ouachrif et al. 2012)
Hexahydroxydiphenic acid ^PJ^											X				(Kasimsetty et al. 2010)
Glycosides ^PL^			X	X											(Das and Barman 2012)
λ-tocopherol ^PS^												X			(Bishayee et al. 2011)
Quinones ^PP^					X		X								(Ouachrif et al. 2012; Setiadhi and Sufiawati 2017)
Saponins ^PP^					X		X								(Ouachrif et al. 2012; Govindappa 2015)
Sterols ^PS^											X				(Bishayee et al. 2011)

PP, Peel; PJ, juice; PS, seed; PF, flower; PL, leaves and NS, Not Specified part

In general, the most essential pomegranate compounds can be divided into two major groups: phenolic compounds and fatty acids.

*Phenolic compounds* consist of a hydroxyl group (–OH), known as phenol, bonded to an aromatic hydrocarbon group as a common part of their structure. Based on the number of phenols in the molecules, the compounds can be simple phenols or polyphenols. As shown in Table 1, there are many different types of phenolic compounds in all parts of the pomegranate tree. Hydrolyzable tannins and flavonoids are the most important subgroups.

*Hydrolyzable tannins* are polyphenolic substances derived from gallic acid (*3, 4, 5-trihydroxybenzoic acid*), including gallotannins and ellagitannins. The most abundant type of ellagitannin is punicalagin. While punicalagin can be hydrolyzed into smaller phenolic compounds such as ellagic acid, the gallotannins can be hydrolyzed into gallic acid.

*Ellagic acid* is an important phenolic acid in pomegranate fruit. It can be found everywhere in the fruit such as the juice, peel, and seeds, among other unspecified pomegranate parts (Table 1), and is responsible for the highest number of bioactivities. Pomegranate ellagic acid has been reported to have positive effects against breast cancer, colon cancer, prostate cancer, and hepatocarcinogenesis (Bishayee et al. 2013; Ahmed et al. 2017; Tamamm et al. 2018; Mansoury 2019). It has also been shown to have anti-allergic, anti-diabetic, anti-inflammatory, antimicrobial, and anti-tyrosinase activity, and has also been linked to the protection against Alzheimer’s Disease (AD) (Panichayupakaranant et al. 2010; Kar et al. 2011; Kerimi et al. 2017; Nirwana 2018; Suman and Bhatnagar 2019; Zehra et al. 2019).

The broad presence of ellagic acid in all parts of the fruit may indicate that all types of pomegranate consumers would benefit from its bioactive capacity. However, ellagic acid absorption may be controversial. Seeram et al. (2004) showed a rapid increase of ellagic acid in human plasma, reaching maximum levels in the plasma after 1 h of pomegranate juice consumption. Nevertheless, in another study (Cerdá et al. 2006), no polyphenols were detected in the plasma or urine of the human patients after pomegranate juice ingestion.

However, the ellagitannins bioavailability can be considered poor mainly due to their low solubility in gastric environments, limited intestinal absorption among other factors (González-Sarrías et al. 2015). Recent reports indicate that the ellagitannins biological effects are associated with its derivate metabolites: the urolithins (urolithin A, B, C and D) (Tomás-Barberán et al. 2017). The urolithins result of the ellagitannins transformation through lactone-ring cleavage, decarboxylation and de-hydroxylation reactions by intestinal microbiota (García‐Villalba et al. 2020). Although numerous studies indicate the positive health effects of pomegranate urolithins, most of the both in vitro and in vivo studies (animal and human) do not specify the pomegranate variety used in the trial (Larrosa et al. 2010; Yuan et al. 2016; Les et al. 2018; Mazumder et al. 2019; Kujawska et al. 2019). The pomegranate cultivar omission can compromise the homogeneity and replicability of the tests, since the ellagitannins content depends of several factors such as the pomegranate variety, the fruit part used, the extraction method, etc. (García-Villalba et al. 2015).On the other hand, similar to ellagic acid, but only with one phenol ring, we find *gallic acid*. In pomegranates, gallic acid is found mainly in the peel and juice (Table 1). The bioavailability of this compound seems to be high, and its absorbability fast and good (Lafay and Gil-Izquierdo 2008). Also, some scientific works have indicated that gallic acid bioavailability from pomegranates, in the shape of food or as a pure compound, seems to be similar (Shahrzad and Bitsch 1998; Shahrzad et al. 2001). This favors the independence of the food matrix for the bioavailability of acid gallic, and therefore, dietary supplementation from industrially-produced pomegranate compounds may be as effective as fruit consumption, at least regarding this particular compound. In general, gallic acid has been observed to have anti-diabetic, antimicrobial and anti-tyrasianel activity, and positive effects against colon cancer and hepatocarcinogenesis (Kasimsetty et al. 2010; Bishayee et al. 2013; Hosseini et al. 2016; AlMatar et al. 2019).

*Flavonoids* are a large group of secondary plant metabolites defined by a diphenylpropane structure and categorized as polyphenolic compounds (Gullon et al. 2016). In pomegranate trees, flavonoids are present in the peel, leaves, and juice. The main pomegranate flavonoids are catechin, epicatechin, kaempferol, quercetin, luteolin, rutin, and anthocyanins (cyanidin and delphinidin). From the number of bioactivities point of view, the most relevant seems to be luteolin (Table 1).

Anthocyanins are responsible for the red color of the pomegranate fruit and its seeds, from which the juice is obtained; this color depends on the type of anthocyanin and its concentration, where the delphinidin derivatives are responsible for the blue and violet color, while pelargonidin is related to the orange-red color (Harborne 1982). All of these compounds have high antioxidant activities. Commercial pomegranate juices show an antioxidant activity that is three times greater as compared to red wine. The main antioxidant compounds in pomegranate juice are hydrolyzable tannins (around 10% of the total antioxidant activity), but anthocyanidins and ellagic acid derivatives also contribute to the pomegranate’s total antioxidant capacity (Melgarejo-Sánchez et al. 2015; Gil et al. 2000). In this sense, it can be confirmed that pomegranate juice is one of the beverages with the most antioxidant capacity in the following order: pomegranate juice > red wine > grape juice > blueberry juice > blackberry juice = juice of lingonberries > orange juice = cold tea drinks = apple juice (Seeram et al. 2008).

*Luteolin* is a type of flavonoid that is characterized by a double bond between C2 and C3. It is one of the most common flavonoids and is mainly found in pomegranate juice, and due to this, it could be established that pomegranate juice and fresh fruit consumers will benefit from its bioactive capacity. Pomegranate luteolin has been shown to have anti-cancer activity due to its interference of cancer metastasis, suppression of cell growth, increase of cell adhesion, inhibition of cell migration, and suppression of chemotaxis towards the proteins involved (Rocha et al. 2012). It has also been shown to inhibit the progression of prostate cancer (Wang et al. 2012). Lastly, some research studies indicate that luteolin has antimicrobial and anti-inflammatory activity (Prithviraj 2018).

Some studies suggest that luteolin may be quickly absorbed (with the highest peak level after 1 h of ingestion) and slowly eliminated, thereby demonstrating the possibility of accumulation in the body (Chen et al. 2006). On the other hand, as with gallic acid, it has been suggested that the bioavailability of luteolin may be higher when ingested in food form when compared with the pure compound (Zhou et al. 2007). This favors the hypothesis that pomegranate fruit consumption may be healthier than the industrially produced supplements that contain pomegranate compounds.

Another equally important pomegranate biocompound are *Fatty Acids*. A fatty acid is a carboxylic acid with a long chain that can either be saturated or unsaturated. Almost all fatty acids found in food have an even number of carbon atoms in an unbranched chain conformation (Coultate 2009). Pomegranates are especially interesting due to the composition of their essential fatty acids (linoleic, linolenic and arachidonic, punicic acid). These are mainly polyunsaturated fatty acids, and play an important role in the prevention of cardiovascular diseases, among other heart problems (Grande 1988; De Hoya and Mata 1989). Among others, the major pomegranate fatty acid studied is punicic acid (Mphahlele et al. 2017).

*Punicic acid* can be described as a polyunsaturated fatty acid and a conjugated α-linolenic acid. It is mainly found in pomegranate seed oil (up to 95% of the total amount of fatty acids) (Arun and Singh 2012), and this is why it was named after *Punica granatum*. In addition, it may be found in small amounts in pomegranate juice, perhaps added during the extraction process. Preclinical studies indicated that punicic acid interfere in the metastasis of breast cancer, having effects against prostate cancer, and showing anti-diabetic, anti-oxidant, and anti-inflammatory activities (Verma et al. 2010; Wang et al. 2012; Rocha et al. 2012; Banihani et al. 2014; Sahebkar et al. 2016). These positive results are being studied and confirmed in several clinical trials with humans; however, in these cases, as a common rule, the pomegranate varieties are not specified (Mirmiran et al. 2010; Asghari et al. 2012).

Based on this evidence, it could be established that the intake of punicic acid by fresh pomegranate consumers may be high because of the ingestion of the whole seeds, in contrast with pomegranate juice consumers, as the content of this acid may be lower and, therefore, may not benefit from its bioactive capacity.

Studies regarding the absorption of punicic acid in animals have shown that it is slowly absorbed in an unchanged state, while part of it may be quickly converted to Conjugated Linoleic Acid (CLA) (Tsuzuki et al. 2006), with both of these compounds found in tissues and plasma 24 h after ingestion (Yuan et al. 2009). CLA is important, because it has been attributed to several positive health benefits (Lopez-Lazaro 2009). In a 28-day study in humans, PA was also partially converted to CLA, and the authors suggested the possibility that ePA or the derived CLA could induce lipid peroxidation in humans (Yuan et al. 2009).

Even though pomegranate punicic acid has been attributed to having several bioactivities, their mechanisms of action are unclear, and therefore, the bioactive capacity associated with punicic acid may be due to its conversion into conjugated linoleic acid. In this sense, punicic acid would be a precursor of the bioactive compounds and related bioactivities, and more studies are needed to fully understand the underlying mechanisms.

### Pomegranate bioactivity potential

As mentioned above, from the scientific point of view, the term "bioactive" is a synonym of "biologically active". In that sense, the bioactivity of an element could be represented by one, or more, substances that have biological activity, that is, it causes a specific effect, response and/or reaction (Abdelkarim et al. 2014).

The potential bioactivity of pomegranate has been traditionally attributed to the exceptional antioxidant activity of the fruit (Glazer et al. 2012). In the last few years, the total antioxidant capacity of different varieties has been reported by several studies as part of their characterization process (Hmid et al. 2017, 2018; Silva et al. 2019).

The total antioxidant capacity of pomegranate reflects the total amount of antioxidant compounds, but it does not specify their nature. For instance, a variety with a high total antioxidant capacity could be missing the specific compound responsible for the mechanism that improves type II diabetes, while a low total antioxidant capacity variety could have the necessary compound (Dludla et al. 2018). Therefore, the total antioxidant capacity will be given little importance in the present report. The potential bioactivity of pomegranate is highly dependent on the variety and the part of the fruit due to the substantial variability of compounds found in each variety and part of the plant. Thus, the present article focuses on these.

### Pomegranate varieties

Even though pomegranate has been widely studied, few studies have compared the level of intensity of an activity (i.e., anti-diabetic, or anti-breast cancer) among different varieties. In some studies, the variety has been unspecified, giving it little importance. It is possible that the most common commercial varieties are used, although this is unknown. The phytochemical composition of many plants has changed over time due to domestication (Holland and Bar-Ya’akov 2018). Therefore, it seems logical to find different nutraceutical effects among varieties. In fact, when the phenolic profile of different pomegranate varieties were studied at the same time, statistical differences were found (Di Stefano et al. 2019). It has also been proven that health effects (bioactive compounds content) and attractiveness factors (color, size, flavor) of pomegranate fruit are not correlated with each other, and will vary with variety and season (Hmid et al. 2017; Derakhshan et al. 2018).

Pomegranate varieties can be divided into 3 groups: sweet, sweet–sour, and sour varieties (Watson and Preedy 2013), and this classification depends on the sugar:acid ratio (Hmid et al. 2018). Whether a variety is sweet or sour is not given any importance in the clinical studies on the bioactive capacities of pomegranate. The sweet and sour varieties have been compared for a certain bioactivity only in very few occasions. Sour varieties have been found to have greater bactericidal effects than the sweet ones (Fazeli et al. 2011; Naziri et al. 2012). However, due to the lack of studies, it is difficult to conclude whether there are differences or not in the bioactive capacity between sweet, sweet–sour, and sour varieties or if it is just a variability between varieties.

Between the varieties with bioactive capacity mentioned in the preclinical studies from the last decades, a high diversity was observed, as shown in Table 2, where it is possible to observe the different bioactive capacities of varieties and if the studies were carried out in vitro or in vivo (animal and human models). Note that, as in the identification of the bioactive compounds present in the different pomegranate parts (Table 1), most of the studies are carried out in vitro, and among the in vivo studies, only a small portion are studies with human models.

**Table 2:** Bioactive among different pomegranate parts and varieties. Alphabetically ordered according to variety

Variety	Peel	Fruit	Juice	Seed	Flower	Leaves	Bioactivity	In vitro	In vivo		References
									Animals	Humans	
Acide	X						+	X			(Abid et al. 2017)
Amrouz	X						+		X		(Ouachrif et al. 2012)
Anar	X						+			X	(Kamali et al. 2015)
Arakta	X						+	X			(Fawole et al. 2012)
Badana	X						+	X			(Khalil et al. 2017)
Bhagwa			X				+ +	X			(Opara et al. 2009)
			X						X		(Husain et al. 2018)
	X							X			(Fawole et al. 2012)
Borde de Albatera	X						+	X			(Rosas-Burgos et al. 2017)
Borde de Beniel	X						+	X			(Rosas-Burgos et al. 2017)
Chetoui					X		+ +	X			(Bekir et al. 2016)
					x			X			(Bekir et al. 2013b)
Daqingpi						X	+	X			(Zhang et al. 2010)
Dente di Cavallo				X			+ +	X			(Lucci et al. 2015)
	X	X	X					X			(Altieri et al. 2019)
Desi	X						+	X			(Khalil et al. 2017)
Egypt			X				+	X			(Opara et al. 2009)
Espagnoule					X		+	X			(Bekir et al. 2016)
Gabsi	X						+ + + +	X			(Abid et al. 2017)
					X			X			(Bekir et al. 2016)
	X							X			(Kharchoufi et al. 2018a)
	X			X	X	X		X			(Elfalleh et al. 2012)
						X		X			(Bekir et al. 2013a)
						X		X			(Bekir et al. 2013a)
	X							X			(Mansour et al. 2013)
Ganesh	X						+ +	X			(Malviya et al. 2014)
	X							X			(Fawole et al. 2012)
Garsi					X		+ +	X			(Bekir et al. 2016)
					x			X			(Bekir et al. 2013b)
Helow		X					+		X		(Subash et al. 2014)
Hicaznar	X						+	X			(Rosas-Burgos et al. 2017)
Indian white			X				+	X			(Opara et al. 2009)
Kabul			X				+	X			(Vaithiyanathan et al. 2011)
Kandhari	X						+	X			(Khalil et al. 2017)
Mollar de Elche	X	X	X				+ + +	X			(Altieri et al. 2019)
	X							X			(Gullon et al. 2016)
	X							X			(Fawole et al. 2012)
	X							X			(Rosas-Burgos et al. 2017)
Nana	X			X	X		+		X		(Wafa et al. 2017)
Nebli	X						+	X			(Abid et al. 2017)
Omani			X				+ +		X		(Subash et al. 2015)
			X					X			(Opara et al. 2009)
Piñón Tierno de Ojós	X						+	X			(Rosas-Burgos et al. 2017)
Rabbab-e Neyriz			X				+	X			(Bazargani-Gilani et al. 2015)
			X							X	(Barati Boldaji et al. 2020)
Rafrafi					X		+	X			(Bekir et al. 2016)
Ruby	X						+ +	X			(Fawole et al. 2012)
	X							X			(Arun et al. 2017)
			X					X			(Opara et al. 2009)
Sefri	X						+		X		(Ouachrif et al. 2012)
Shishe Kab	X			X		X	+	X			(Tehranifar et al. 2011)
Tounsi	X						+	X			(Abid et al. 2017)
Valenciana de Albatera	X						+	X			(Rosas-Burgos et al. 2017)
Wonderful	X						+ + + + + + +	X			(Martínez et al. 2019)
	X							X			(Glazer et al. 2012)
	X								X		(Ashoush et al. 2013)
								X			(Verotta et al. 2018)
			X					X			(Velagapudi et al. 2016)
	X								X		(Mastrogiovanni et al. 2019)
	X								X		(Gupta et al. 2019)
	X							X			(Shirode et al. 2015)
			X					X			(Gil et al. 2000)
		X								X	(Heber et al. 2007)
	X		X						X		(Al-Jarallah et al. 2013)
		X							X		(Hartman et al. 2006)
		X	X					X			(Kim et al. 2002)
		X	X						X		(Seeram et al. 2007)
			X							X	(Pantuck et al. 2006)
			X					X			(Adams et al. 2006)
	X		X	X				X			(Lansky et al. 2005)
	X		X	X				X			(Aslam et al. 2006)
			X					X			(Seeram et al. 2005)
	X				X				X		(Aviram et al. 2008)
	X							X			(Shirode et al. 2014)
			X							X	(Sumner et al. 2005)
			X					X			(Velagapudi et al. 2016)
			X					X			(Khateeb et al. 2010)
	X							X			(Fawole et al. 2012)
	X								X		(Morzelle et al. 2016)
			X					X			(Kasimsetty et al. 2010)
			X							X	(Bookheimer et al. 2013)
			X							X	(Siddarth et al. 2020)
			X						X		(Makino-Wakagi et al. 2012)
			X					X	X		(Rojanathammanee et al. 2013)
Zaghwani					X		+ +	X			(Bekir et al. 2016)
					X			X			(Bekir et al. 2013b)
Zehri					X		+	X			(Bekir et al. 2016)

However, aware of the human studies are limited and based on the studies available, it was possible to define a bioactivity range according to the number and intensity of activities found aiming to identify the pomegranate varieties more used and/or studied. In this sense, the most bioactive varieties identified, in terms of the number of scientific studies, were Wonderful > Gabsi > Mollar de Elche > Ganesh > Ruby. As for the type of tests conducted, it is possible to highlight that for the Wonderful variety, almost half of the studies were carried out in vivo (26% clinical studies), while for Gabsi, Mollar de Elche, Ganesh, and Ruby, these were in vitro. The same trend was observed for the varieties with the least bioactivity, where the studies were carried out predominantly in vitro.

Nevertheless, this predominance of preclinical studies (in vivo and in vitro) over clinical trials in humans is reversed when were considered studies in which the pomegranate variety was not specified (Table 3). In most cases, the references on the article for the pomegranate variety was simply "acquired at the local market". Considering that a significant proportion of these studies were carried out in vivo, and even with humans, it can be affirmed and highlighted an important mistakes and deficiencies of these studies, since it can imply serious difficulties for future replications and/or standardization of results based on the compositional pomegranate varieties variations.

**Table 3:** Bioactive among different pomegranate part in unspecified pomegranate variety

Variety	Pomegranate part						Experimental			References
	Peel	Fruit	Juice	Seed	Flower	Leaves	In vitro	In vivo		
								Animals	Humans	
Unspecified	X			X			X			(Heena et al. 2018)
	X						X			(Liu et al. 2014)
	X						X			(Du et al. 2019)
	X								X	(González-Sarrías et al. 2015)
			X						X	(Bookheimer et al. 2013)
			X						X	(Banihani et al. 2019)
			X						X	(Sohrab et al. 2019)
			X						X	(Fuster-Muñoz et al. 2016)
			X						X	(González-Ortiz et al. 2011)
				X					X	(Asghari et al. 2012)
				X					X	(Mirmiran et al. 2010)
	X						X			(Deng et al. 2017)
	X							X		(Al-Megrin 2017)
			X					X		(Kujawska et al. 2019)
						X	X			(Li et al. 2016)
			X				X			(Les et al. 2018)
			X						X	(Rosenblat et al. 2006)
			X					X		(Kaplan et al. 2001)
	X						X			(Arunkumar and Rajarajan 2018)
		X						X		(Syeda et al. 2018)
	X						X			(Hanani et al. 2019)
	X						X			(Kharchoufi et al. 2018b)
				X				X		(Gabizon et al., 2019)
	X							X		(González-Trujano et al. 2015)
	X							X		(Kumar et al. 2013)
					X			X		(Mithun et al. 2012)
					X			X		(Nasiri et al. 2017)
	X					X		X		(Janardan Salwe et al. 2014)
		X						X		(Yuniarti et al. 2018)
	X						X			(Sudheesh et al. 2018)
						X	X			(Elbatanony et al. 2019)
				X			X			(Al-Huqail et al. 2018)
			X						X	(Matthaiou et al. 2014)
			X						X	(Achraf et al. 2018)
			X						X	(Davidson et al. 2009)
		X							X	(Hosseini et al. 2016)
						X	X			(Giamogante et al. 2018)
			X						X	(Sohrab et al. 2019)
	X						X			(Panichayupakaranant et al. 2010)
	X		X				X			(Pagliarulo et al. 2016)
	X							X		(Althunibat et al. 2010)
		X			X	X	X			(Kiraz et al. 2016)
				X			X			(Mandal et al. 2017)
	X							X		(Larrosa et al. 2010)
					X		X			(Durgawale and Datkhile 2016)
	X						X			(Šavikin et al. 2018)
			X					X		(Aharoni et al. 2015)
	X						X			(Al-Bahadily et al. 2019)
	X	X	X				X			(Altieri et al. 2019)

In addition, Table 4 shows the specific bioactivities related to each variety, but also for unspecified varieties, aiming to maintain the global view both of the bioactive compounds in pomegranate and varietal identification as a relevant part of the study.

**Table 4:** Specific bioactivities linked to pomegranate varieties and pomegranate parts as peel (PP), juice (PJ), seed (PS), flower (PF) and leaves (PL)

		Related activities												References
Variety	Pomegranate part	Medical	Hepatoprotetive	Cardioprotetive	Antinociceptive	Anti-diabetic	Antioxidant	Antimicrobial	Antifungal	Neurodegenerative	Anti-allergic	Anti-cancer	Anti-inflamatory	
Acide	PP						X							(Abid et al. 2017)
Amrouz	PP				X								X	(Ouachrif et al. 2012)
Anar	PP												X	(Kamali et al. 2015)
Arakta	PP	X					X	X						(Fawole et al. 2012)
Badana	PP						X							(Khalil et al. 2017)
Bhagwa	PJ PP	X					X	X						(Opara et al. 2009; Fawole et al. 2012; Husain et al. 2018)
Borde de Albatera	PP							X	X					(Rosas-Burgos et al. 2017)
Borde de Beniel	PP							X	X					(Rosas-Burgos et al. 2017)
Chetoui	PF					X	X			X		X	X	(Bekir et al. 2013b, 2016)
Daqingpi	PL						X							(Zhang et al. 2010)
Dente di Cavallo	PP PF PJ	X					X					X		(Lucci et al. 2015; Altieri et al. 2019)
Desi	PP						X							(Khalil et al. 2017)
Egypt	PJ							X						(Opara et al. 2009)
Espagnoule	PF					X				X				(Bekir et al. 2016)
Gabsi	PP PS PF PL	X				X	X	X		X			X	(Elfalleh et al. 2012; Mansour et al. 2013; Bekir et al. 2013a, 2016; Abid et al. 2017; Kharchoufi et al. 2018a)
Ganesh	PP	X					X	X						(Fawole et al. 2012; Malviya et al. 2014)
Garsi	PF PF					X	X			X		X	X	(Bekir et al. 2013b, 2016)
Helow	PJ						X		X					(Subash et al. 2014)
Hicaznar	PP							X	X					(Rosas-Burgos et al. 2017)
Indian white	PJ							X						(Opara et al. 2009)
Kabul	PJ							X						(Vaithiyanathan et al. 2011)
Kandhari	PP						X							(Khalil et al. 2017)
Mollar de Elche	PP PJ PF	X					X	X	x					(Fawole et al. 2012; Gullon et al. 2016; Rosas-Burgos et al. 2017; Altieri et al. 2019)
Nana	PP PS PF							X						(Wafa et al. 2017)
Nebli	PP						X							(Abid et al. 2017)
Omani	PJ							x		X				(Opara et al. 2009; Subash et al. 2015)
Piñón Tierno de Ojós	PP							X	X					(Rosas-Burgos et al. 2017)
Rabbab-e Neyriz	PJ							X						(Bazargani-Gilani et al. 2015)
Rafrafi	PF					X				X				(Bekir et al. 2016)
Ruby	PP PJ	X		X		X	X	X						(Opara et al. 2009; Fawole et al. 2012; Arun et al. 2017)
Sefri	PP				x								X	(Ouachrif et al. 2012)
Shishe Kab	PP PS PL						X		X					(Tehranifar et al. 2011)
Tounsi	PP						X							(Abid et al. 2017)
Unspecified	PP PS						X							(Heena et al. 2018)
Unspecified	PP PF												X	(Syeda et al. 2018; Du et al. 2019)
Unspecified	PP PL											X		(Li et al. 2016; Deng et al. 2017)
Unspecified	PP	X				X	X	X	X	X			X	(Althunibat et al. 2010; Kumar et al. 2013; Al-Megrin 2017; Šavikin et al. 2018; Arunkumar and Rajarajan 2018; Kharchoufi et al. 2018b; Sudheesh et al. 2018; Al-Bahadily et al. 2019; Hanani et al. 2019)
Unspecified	PJ	X		X		X	X							(Kaplan et al. 2001; Rosenblat et al. 2006; Davidson et al. 2009; Sohrab et al. 2019)
Unspecified	PS									x		X	X	(Mandal et al. 2017; Gabizon et al. 2019)
Unspecified	PP PF PL				X								X	(Mithun et al. 2012; Janardan Salwe et al. 2014; González-Trujano et al. 2015)
Unspecified	PF	X												(Nasiri et al. 2017; Yuniarti et al. 2018)
Unspecified	PS PJ						X							(Matthaiou et al. 2014; Al-Huqail et al. 2018)
Unspecified	PJ PF	X											X	(Hosseini et al. 2016; Achraf et al. 2018)
Unspecified	PL	X												(Giamogante et al. 2018)
Unspecified	PP							X			X		x	(Panichayupakaranant et al. 2010)
Unspecified	PP PS PL							X						(Pagliarulo et al. 2016; Elbatanony et al. 2019)
Unspecified	PP PF PL	X					X					X		(Durgawale and Datkhile 2016; Kiraz et al. 2016; Altieri et al. 2019)
Unspecified	PP PJ												X	(Larrosa et al. 2010; Aharoni et al. 2015)
Valenciana de Albatera	PP							X	X					(Rosas-Burgos et al. 2017)
Wonderful	PP PJ PF PS	X	X	X		x	X	X	X	X		X	X	(Gil et al. 2000; Kim et al. 2002; Lansky et al. 2005; Seeram et al. 2005, 2007; Sumner et al. 2005; Adams et al. 2006; Pantuck et al. 2006; Aslam et al. 2006; Hartman et al. 2006; Heber et al. 2007; Aviram et al. 2008; Kasimsetty et al. 2010; Khateeb et al. 2010; Glazer et al. 2012; Makino-Wakagi et al. 2012; Fawole et al. 2012; Al-Jarallah et al. 2013; Rojanathammanee et al. 2013; Ashoush et al. 2013; Shirode et al. 2014, 2015; Velagapudi et al. 2016; Morzelle et al. 2016; Verotta et al. 2018; Mastrogiovanni et al. 2019; Gupta et al. 2019; Martínez et al. 2019)
Zaghwani	PF					X	X			X		X	X	(Bekir et al. 2013b, 2016)
Zehri	PF					X				X				(Bekir et al. 2016)

PP, Peel; PJ, juice; PS, seed; PF, flower and PL, leaves

### Pomegranate parts

In general, the pomegranate fruit is comprised by the pericarp, mesocarp and seeds arranged in eight carpels superimposed in two whorls and protected by the carpelar membranes (Gilg and Schürhoff 1959; Strasburger et al. 1986). Based on the bibliographical review, when the bioactive compounds and nutraceutical value of pomegranate fruits are studied, the pericarp or skin is commonly named peel (PP). The PP could comprise up to a maximum of 50% of the total fruit weight, and it is an important source of bioactive compounds (Viuda-Martos et al. 2010). In Spanish pomegranate varieties, Melgarejo (1993) determined that the pomegranate seeds comprised about 60–70% of the total weight, while the PP remained in the 25–40% range (Melgarejo 1993).

The seeds are the edible part of the fruit, and are formed by a hard inner part, which contains the cotyledons, the embryo, and the testa, a pulpy membrane from which the juice (PJ) is extracted. In most of the published articles on pomegranate seeds, the authors focused on the hard inner part of the seed without taking the pulpy membrane into consideration.

Accordingly, to facilitate the comprehension of this review, the commonly used terminology for the pomegranate parts, although botanically inaccurate (Gilg and Schürhoff 1959; Melgarejo and Salazar 2003), was maintained. Therefore, in the presen review, the term pomegranate seed (PS) will be used only for the hard inner part of the seed. These differences in the terminology of the pomegranate fruit parts can be due to the objective of the articles consulted, which were more focused on the biochemical part than the botanical one, but could also be related with the area of training and research of the authors (pharmaceutical, nutritional, medical, etc.).

Although the fruit parts were the most commonly used plant part on the bibliography, other parts of the pomegranate tree have also been used, such as the pomegranate leaves (PL) and flowers (PF). Even though the literature shows that every part of pomegranate has been used in several scientific assays, a clear predominance of studies employing pomegranate peel and juice was observed (Tables 2 and 3).

The identification of the bioactive capacity of the different parts of the fruit is greatly important, as different consumers have different eating habits. However, it also affects the form and the mode in which its use could be enhanced in different sectors such as the pharmaceutical, food, or cosmetic sectors, among others. Based on the evidence found in Tables 2 and 3, it could be confirmed that in both specified and unspecified pomegranate varieties, the PP was the most studied bioactive part of the fruit. It was followed by PJ, PS, and other parts (PL and PF), perhaps due to the high content of biological compounds found in this part of the fruit (Orak et al. 2012).

### Pomegranate peel (PP)

Thus, in terms of bioactivity, the PP was the most studied part of the fruit. Based on the review of the literature, it was observed that the antioxidant activity of PP was the most widely studied for all pomegranate varieties. This was followed by antimicrobial, anti-inflammatory, and antifungal activities, among others.

Antioxidant capacity provides, in addition to health and medical applications, and mainly due to its influence on the tyrosinase-inhibitor mechanism, the suitable characteristics for use in other sectors such as food preservation, development of functional dietary food, and agriculture improvement (Tehranifar et al. 2011; Khalil et al. 2017; Abid et al. 2017; Heena et al. 2018; Kharchoufi et al. 2018a; Šavikin et al. 2018; Altieri et al. 2019).

In addition, the pomegranate pericarp has proven to be effective against many types of bacteria. The compound isolated from the PP have been shown to be effective against Gram-negative bacteria (*Escherichia coli, Salmonella sp, Pseudomonas aeruginosa, P. putida, Enterobacter aerogenesand, Klebsiella pneumonia*), and Gram-positive bacteria (B*acillus subtilis, Listeria innocua, L. monocytogenes, Taphylococcus aureus,* and *Staphylococcus aureus*) (Wafa et al. 2017; Kharchoufi et al. 2018a). In one experiment, PP extracts also showed effects in preserving meat against 8 different strains of *Listeria monocytogenes* (Hayrapetyan et al. 2012). In the mentioned study, the various extracts had different strengths, attributed to variability in the raw materials (different varieties). In a complementary manner, antifungal (*Penicillium digitatum, P. italicum, Botrytis cinerea, Rhizopus stolonifer,* and *Saccharomyces cerevisiae*) (Kharchoufi et al. 2018a) and antivirus (HSV-2) (Arunkumar and Rajarajan 2018) activities were also reported.

The anti-inflammatory and antinociceptive activity of PP extract has been verified through different manners of administration: oral, gel, nano-emulsions, intraperitoneal, and intra-cerebroventricular (Lansky and Newman 2007; Janardan Salwe et al. 2014; González-Trujano et al. 2015; Nirwana 2018). This is in agreement with other studies regarding in vivo and in vitro assays (Verotta et al. 2018; Sudheesh et al. 2018; Mastrogiovanni et al. 2019). An extract of the whole pericarp also showed anti-inflammatory activity in damaged mice and albino rats (Syeda 2018; Wang et al. 2014).

Regarding the anti-cancer activity, PP extract showed suppressive effects on two types of human cancer, prostate cancer (Deng et al. 2017) and breast cancer (Shirode et al. 2015), and also on stimulated apoptosis. It could be highlighted that the last studies indicated that the growth inhibition of cancer cells must not only be attributed to the high antioxidant capacity of PP, as the extract may also be involved in DNA repair processes and induction of double-strand breaks. However, both the knowledge of the anticancer specific mechanism and its efficacy in humans continues to be limited, since the majority studies that indicate these activities normally are carried out using cell models.

In a study from 2010 (Olapour and Najafzadeh 2010), an antiepileptic effect of pomegranate was suggested. In that study, mice were administrated PP extract in doses of 100, 200, 400, and 600 mg/kg. Then, they were injected with strychnine, which is a competitive antagonist of the inhibitory neurotransmitter glycine at specific receptors, used to kill rodents causing muscular convulsions and death due to asphyxia. The PP extract administered had a significant anticonvulsive effect on the treated mice, and the animals suffered a lesser number of convulsions, the convulsions lasted less, and they lived for longer.

### Pomegranate juice (PJ)

The PJ is the second pomegranate part with the largest number of research studies. PJ has also been linked to microbial inhibiting properties with interesting medical (Altieri et al. 2019; Silva et al. 2019) and industrial applications. Some works have demonstrated that dipping chicken in PJ reduced microbial growth under refrigeration temperatures (Bazargani-Gilani et al. 2015). Along the same line, other research studies have shown that the growth of certain Gram-positive bacteria such as *Escherichia coli, Listeria monocytogenes, Staphylococcus aureus, Bacillus cereus, and Clostridium perfringens* and Gram-negative bacteria such as *Helicobacter pylori* and *Vibrio parahemolyticus*, were inhibited by PJ (Pagliarulo et al. 2016; Juneja et al. 2016). The inhibition of *Helicobacter pylori* may indicate that PJ could be useful as a supplement for treating gastric ulcers, which are caused by this organism. However, *Escherichia coli*, inhibited by PP extract, has been shown to be unaffected by PJ in another study (Haghayeghi et al. 2013). Nevertheless, in another experiment, PJ seemed to inhibit the growth of *Escherichia coli* (Pagliarulo et al. 2016; Juneja et al. 2016). This controversy supports the suggestion that different pomegranate varieties are likely to have different bioactive compounds. Therefore, when considering PJ as a preservative or as a supplement against certain diseases, more in-depth research of the specific variety must be carried out first.

Moreover, cell models studies indicated that PJ interferes in metastasis of breast cancer, suppressing cell growth, increasing cell adhesion, inhibiting cell migration, and suppressing chemotaxis of proteins involved in breast cancer metastasis (Rocha et al. 2012). Prostate cancer and colon cancer progression were also inhibited by PJ as with breast cancer (Kasimsetty et al. 2010; Wang et al. 2012). Another study conducted using cell model reported that the intestinal bacterial metabolites resulting from PJ ingestion (urolithins) may play an essential role in the anticancer activity by inhibiting the initiation and proliferation of colon cancer (Kasimsetty et al. 2010). Nevertheless, although they are positive indications and desirable effects, and as indicated above these results are based on tests conducted with cell models. Thus, the health effects verification and validation must be improved and tested in clinical studies in humans.

Resistin, an adipocytokine, is considered the link between obesity and type II diabetes. A study with the Wonderful variety showed that PJ extract suppresses resistin secretion via a mechanism that may involve the degradation of the intracellular resistin protein in adipocytes (Makino-Wakagi et al. 2012). Another study proved that 3 h after ingestion of PJ, fasting serum glucose and insulin resistance were reduced among type II diabetes patients (Rosenblat et al. 2006; Banihani et al. 2014; Altieri et al. 2019). In addition, PJ consumption showed cardioprotective activity (Sumner et al. 2005; Al-Jarallah et al. 2013) even under clinical trial experimental conditions (Sohrab et al. 2019).

Likewise, PJ also showed representative anti-inflammatory activity in clinical trial conditions (Aharoni et al. 2015; Achraf et al. 2018). Several long-term studies (15 months) in mice and an in vitro assay suggested that supplementation with PJ extract may slow the progression of cognitive and behavioral impairments due to Alzheimer’s Disease (AD) (Subash et al. 2014, 2015; Velagapudi et al. 2016). These effects were mainly found for the Wonderful variety, but also for the Omani variety.

However, based on the review of the literature, a deficient standardization of the methodology utilized for juice production was identified. Despite some works indicating if the juice was obtained manually or automatically, in the majority of cases this was not specified, and this could have an impact on the bioactive proprieties analyzed.

### Pomegranate seed (PS)

In pomegranates, PS are significant sources of fiber (Mandal et al. 2017), although their importance lies mainly on its oil. PS oil has been reported to have a great cancer-fighting potential in the reproductive systems, both in males and females (Jasuja et al. 2012). It has also been reported to inhibit aromatase (Kim et al. 2002), the enzyme that produces estrogen from testosterone 17-β-hydroxysteroid dehydrogenase type 1, which is responsible for the conversion of estrone into estradiol. That enzymatic blockade helps to increase the pomegranate’s ability to prevent the growth of estrogen-dependent breast cancer cells in culture and also minimizes the invasiveness of cancer cells (Mandal et al. 2017). PS oil also prevents the proliferation of certain human prostate cancer lines by changing the cell growth cycle and inducing apoptosis (Lansky et al. 2005; Jasuja et al. 2012; Lucci et al. 2015).

As with the other plant parts mentioned above, the PS oil also has anti-inflammatory activity (Mandal et al. 2017). In a study carried out in rats, it was shown to reduce inflammation at the site of the lesion (Coursodon-Boyiddle et al. 2012).

Antimicrobial activity has also been confirmed by means of assays with dried PS extract in a study with meat pâté, which showed the effectiveness of this extract against different strains of *Listeria monocytogenes* (Hayrapetyan et al. 2012), and liquid PS extract against *Salmonella enterica* (Wafa et al. 2017) and plant pathogenic fungi such as *Penicillium italicum, Botrytis cinerea,* and *Rhizopus stolonifer* (Tehranifar et al. 2011).

Another study carried out in rats (Nekooeian et al. 2014), showed the anti-diabetic activity of PS oil through the improvement of insulin secretion without changing fasting blood glucose. In mice, PS oil ameliorated high-fat diet-induced obesity and insulin resistance, non-aligned with changes in food intake (Shirode et al. 2014). Other studies have confirmed the pharmacological potential of PS through focused trials on its cosmetic applications for the regeneration of the skin (Aslam et al. 2006), and its medical applications through the study of its influence/treatment of neurodegenerative diseases such as Creutzfeldt—Jacob disease (CJD), multiple sclerosis (MS), Parkinson disease (PD), and Alzheimer's disease (AD) (Gabizon et al. 2019).

Among the varieties studied for PS bioactivity we mainly found the Wonderful variety, but also the Dente di Cavallo, Gabsi, Nana, Shishe Kab varieties. The vast majority of the works were conducted with unspecified varieties of pomegranate.

### Pomegranate flower (PF)

Based on the bibliographic results, a significant increase in the interest of the scientific community regarding the applications and bioactivities of the pomegranate flower (PF) was observed in recent years.

PF extracts have been shown to have beneficial effects against diabetes by reducing the fasting blood glucose in rats (Bagri et al. 2009). In another study (Gil et al. 2000), a pomegranate extract made from a mixture of unspecified parts showed beneficial effects in humans with type II diabetes. The extracts antagonized the hyperglycemia-induced oxidative stress, illustrated by the drop in the levels of plasma malondialdehyde and the increase in the total level of plasma glutathione. The PFs of seven pomegranate varieties (Chetoui, Espagnoule, Gabsi, Garsi, Rafrafi, Zaghwani, and Zehri) were also studied in regards to their anti-cholinesterase and anti-hyperglycemic activities, finding significant differences among the evaluated varieties (Bekir et al. 2016).

Antioxidant, anti-inflammatory, and anti-breast cancer activities of Chetoui, Garsi, and Zaghwani varieties have also been investigated (Elfalleh et al. 2012; Bekir et al. 2013b).

### Pomegranate leaves (PL)

Antioxidant and anti-inflammatory activities were the major bioactivities studied in PL (Elfalleh et al. 2012; Bekir et al. 2013a; Janardan Salwe et al. 2014). In addition, many works identified the potential of antimicrobial effects of the PF extract on plant pathogenic fungi (P*enicillium italicum, Botrytis cinerea,* and *Rhizopus stolonifer*) (Tehranifar et al. 2011), for Gram negative bacteria (*Pseudomonas aeruginosa, E. coli, and Salmonella typhimurium*), Gram positive bacteria (*Staphylococcus aureus, Listeria monocytogenes, Enterococcus fecalis,* and *Bacillus cereus*), yeast (*Candida albicans*), and fungi such as *Aspergillus niger* (Elbatanony et al. 2019).

The medical application of PL extract was also studied for cancer treatment and prevention (Kiraz et al. 2016; Li et al. 2016; Giamogante et al. 2018). Another study, carried out with rats, showed anticonvulsant and antianxiety effects in the Maximal electroshock and Pentylenetetrazole-induced seizure models (Sarma and Das 2014).

### Combination of different pomegranates parts

Extracts made from mixtures of different pomegranate parts have different sets of compounds that may result in synergistic effects that are greater than those from single compounds (Seeram et al. 2005; Olapour and Najafzadeh 2010). An emulsion made from the combination of PS oil and an extract from PJ, PP, PL, and PF demonstrated evidence of having a high chemo-preventive effect against experimental hepatocarcinogenesis in rats, resulting in fewer animals with visible hepatocyte nodules and lower nodule multiplicity (Bishayee et al. 2011). According to the same study, this effect was probably due to pomegranate phytoconstituents that utilize antioxidant mechanisms to repeal the oxidative stress provoked during diethylnitrosamine-initiated hepatocarcinogenesis.

Another study showed that the same emulsion reversed the increase of inducible nitric oxide synthase (responsible of generating nitric oxide that contributes to chronic inflammatory reactions) in hepatocellular carcinogenesis, indicating a clear anti-inflammatory effect (Bishayee et al. 2013).

The anti-inflammatory activity of pomegranate extract made from different pomegranate parts showed effects against colon inflammation (Larrosa et al. 2010), due to the anti-inflammatory effects of the metabolites (in particular, urolithin-A).

A study in transgenic mouse (Rojanathammanee et al. 2013) concluded that some compounds of pomegranate extract made from PJ and PS attenuated the nuclear factor of activated T-cells in a reported cell line, decreasing Aβ-stimulated tumor necrosis factor α secretion by murine microglia. This indicates that pomegranate produces anti-inflammatory effects in the brain and that adding pomegranate in the diet may attenuate AD development.

Although there are many more preclinical studies available on the bibliography based on the use of pomegranate extracts with interesting bioactivities, the lack of homogeneity in the extract preparation (pomegranate part used, pomegranate part pre-treatment, additional substances among others.) and no pomegranate variety specification make it difficult, and even limit, their classification and, therefore, their comparison and replication.

## Conclusions

Pomegranate varieties differ in their phytochemical compositions, thereby affecting their bioactive capacity. In most of the clinical studies, the variety was unspecified. Therefore, reproducing those studies may prove to be difficult. The variety Wonderful was the one in which the highest number of bioactivities were found throughout the last few years. Thus, it may be one of the most interesting varieties for the consumer from a health point of view.

The different parts of the pomegranate fruit have diverse bioactive capacities. The PP has the most substantial amount of bioactive compounds. Thus, the health-promoting characteristics attributed to pomegranate may mislead the consumers of fresh pomegranates, who normally discard the PP when eating the fruit. In regard to this, PJ consumers may not benefit from the bioactive compounds present in the PS.

It is, therefore, concluded that the promotion of health benefits attributed to pomegranates should be linked to variety and end-user eating habits.

It is, therefore, recommended that future studies specify the pomegranate varieties and parts utilized, as there is a high variability in the amount of bioactive compounds between them. Aside from this, more in vivo studies that isolate the bioactive compounds should be carried out to clarify the activities of single compounds and their synergistic actions with other compounds.

## Data Availability

Not applicable.

## Abbreviations

WoS: Web of Science
S: Scopus
Pom: Pomegranate
PuGr: Punica granatum
PP: Pomegranate Peel
PJ: Pomegranate Juice
PS: Pomegranate Seeds
PL: Pomegranate Leaves
PF: Pomegranate Flower
AD: Alzheimer’s Disease
CJD: Creutzfeldt–Jacob disease
MS: Multiple sclerosis
PD: Parkinson disease

## References

[CR1] Abdelkarim G, Soumaya B, Naima E (2014). What is a bioactive compound? A combined definition for a preliminary consensus. Int J Nutr Food Sci.

[CR2] Abid M, Yaich H, Cheikhrouhou S (2017). Antioxidant properties and phenolic profile characterization by LC–MS/MS of selected Tunisian pomegranate peels. J Food Sci Technol.

[CR3] Achraf A, Hamdi C, Turki M (2018). Natural pomegranate juice reduces inflammation, muscle damage and increase platelets blood levels in active healthy Tunisian aged men. Alexandria J Med.

[CR4] Adams L, Seeram N, Aggarwal B (2006). Pomegranate juice, total pomegranate ellagitannins, and punicalagin suppress inflammatory cell signaling in colon cancer cells. J Agric Food Chem.

[CR5] Aharoni S, Lati Y, Aviram M, Fuhrman B (2015). Pomegranate juice polyphenols induce a phenotypic switch in macrophage polarization favoring a M2 anti-inflammatory state. BioFactors.

[CR6] Ahmed HH, El-Abhar HS, Hassanin EAK (2017). Punica granatum suppresses colon cancer through downregulation of Wnt/β-Catenin in rat model. Rev Bras Farmacogn.

[CR7] Al-Bahadily D, Shari F, Najm M, Al-Salman H (2019). Antimicrobial activity of the compound 2-piperidinone, N-[4-Bromo-n-butyl]-extracted from pomegranate peels. Asian J Pharm.

[CR8] Al-Huqail AA, Elgaaly GA, Ibrahim MM (2018). Identification of bioactive phytochemical from two Punica species using GC–MS and estimation of antioxidant activity of seed extracts. Saudi J Biol Sci.

[CR9] Al-Jarallah A, Igdoura F, Zhang Y (2013). The effect of pomegranate extract on coronary artery atherosclerosis in SR-BI/APOE double knockout mice. Atherosclerosis.

[CR10] AlMatar M, Var I, Kayar B (2019). Evaluation of polyphenolic profile and antibacterial activity of pomegranate juice in combination with rifampin (R) against MDR-TB clinical isolates. Curr Pharm Biotechnol.

[CR11] Al-Megrin WA (2017). In vivo study of pomegranate (Punica granatum) peel extract efficacy against *Giardia lamblia* in infected experimental mice. Asian Pac J Trop Biomed.

[CR12] Althunibat OY, Al-Mustafa AH, Tarawneh K (2010). Protective role of *Punica granatum* L. peel extract against oxidative damage in experimental diabetic rats. Process Biochem.

[CR13] Altieri F, Cairone F, Giamogante F (2019). Influence of ellagitannins extracted by pomegranate fruit on disulfide isomerase PDIA3 activity. Nutrients.

[CR14] Ambigaipalan P, de Camargo AC, Shahidi F (2016). Phenolic compounds of pomegranate byproducts (outer skin, mesocarp, divider membrane) and their antioxidant activities. J Agric Food Chem.

[CR15] Arun N, Singh D (2012). Punica granatum: a review on pharmacological and therapeutic properties. Int J Pharm Sci Res.

[CR16] Arun KB, Jayamurthy P, Anusha CV (2017). Studies on activity guided fractionation of pomegranate peel extracts and its effect on antidiabetic and cardiovascular protection properties. J Food Process Preserv.

[CR17] Arunkumar J, Rajarajan S (2018). Study on antiviral activities, drug-likeness and molecular docking of bioactive compounds of *Punica granatum* L. to herpes simplex virus - 2 (HSV-2). Microb Pathog.

[CR18] Asghari G, Sheikholeslami S, Mirmiran P (2012). Effect of pomegranate seed oil on serum TNF-α level in dyslipidemic patients. Int J Food Sci Nutr.

[CR19] Ashoush IS, El-Batawy OI, El-Shourbagy GA (2013). Antioxidant activity and hepatoprotective effect of pomegranate peel and whey powders in rats. Ann Agric Sci.

[CR20] Aslam MN, Lansky EP, Varani J (2006). Pomegranate as a cosmeceutical source: pomegranate fractions promote proliferation and procollagen synthesis and inhibit matrix metalloproteinase-1 production in human skin cells. J Ethnopharmacol.

[CR21] Aviram M, Volkova N, Coleman R (2008). Pomegranate phenolics from the peels, arils, and flowers are antiatherogenic: studies in vivo in atherosclerotic apolipoprotein e-deficient (E 0) mice and in vitro in cultured macrophages and lipoproteins. J Agric Food Chem.

[CR22] Bagri P, Ali M, Aeri V (2009). Antidiabetic effect of *Punica granatum* flowers: effect on hyperlipidemia, pancreatic cells lipid peroxidation and antioxidant enzymes in experimental diabetes. Food Chem Toxicol.

[CR23] Banihani SA, Makahleh SM, El-Akawi Z (2014). Fresh pomegranate juice ameliorates insulin resistance, enhances β-cell function, and decreases fasting serum glucose in type 2 diabetic patients. Nutr Res.

[CR24] Banihani SA, Shuaibu SM, Al-Husein BA, Makahleh SS (2019). Fresh pomegranate juice decreases fasting serum erythropoietin in patients with type 2 diabetes. Int J Food Sci.

[CR25] Barati Boldaji R, Akhlaghi M, Sagheb MM, Esmaeilinezhad Z (2020). Pomegranate juice improves cardiometabolic risk factors, biomarkers of oxidative stress and inflammation in hemodialysis patients: a randomized crossover trial. J Sci Food Agric.

[CR26] Bazargani-Gilani B, Aliakbarlu J, Tajik H (2015). Effect of pomegranate juice dipping and chitosan coating enriched with Zataria multiflora Boiss essential oil on the shelf-life of chicken meat during refrigerated storage. Innov Food Sci Emerg Technol.

[CR27] Bekir J, Mars M, Souchard JP, Bouajila J (2013). Assessment of antioxidant, anti-inflammatory, anti-cholinesterase and cytotoxic activities of pomegranate (*Punica granatum*) leaves. Food Chem Toxicol.

[CR28] Bekir J, Mars M, Vicendo P (2013). Chemical composition and antioxidant, anti-inflammatory, and antiproliferation activities of pomegranate (*Punica granatum*) flowers. J Med Food.

[CR29] Bekir J, Cazaux S, Mars M, Bouajila J (2016). In vitro anti-cholinesterase and anti-hyperglycemic activities of flowers extracts from seven pomegranate varieties. Ind Crops Prod.

[CR30] Bishayee A, Bhatia D, Thoppil RJ (2011). Pomegranate-mediated chemoprevention of experimental hepatocarcinogenesis involves Nrf2-regulated antioxidant mechanisms. Carcinogenesis.

[CR31] Bishayee A, Thoppil RJ, Darvesh AS (2013). Pomegranate phytoconstituents blunt the inflammatory cascade in a chemically induced rodent model of hepatocellular carcinogenesis. J Nutr Biochem.

[CR32] Bookheimer SY, Renner BA, Ekstrom A (2013). Pomegranate juice augments memory and fMRI activity in middle-aged and older adults with mild memory complaints. Evidence-based Complement Altern Med.

[CR33] Cerdá B, Soto C, Albaladejo MD (2006). Pomegranate juice supplementation in chronic obstructive pulmonary disease: a 5-week randomized, double-blind, placebo-controlled trial. Eur J Clin Nutr.

[CR34] Chen T, Li L-P, Xin-Yan Lu (2006). Absorption and excretion of luteolin and apigenin in rats after oral administration of chrysanthemum morifolium extract. J Agric Food Chem.

[CR35] Coultate T (2009). Food: the chemistry of its components.

[CR36] Coursodon-Boyiddle CF, Snarrenberg CL, Adkins-Rieck CK (2012). Pomegranate seed oil reduces intestinal damage in a rat model of necrotizing enterocolitis. Am J Physiol Liver Physiol.

[CR37] Czieczor L, Bentkamp C, Damerow L, Blanke M (2018). Non-invasive determination of the quality of pomegranate fruit. Postharvest Biol Technol.

[CR38] Das S, Barman S (2012). Antidiabetic and antihyperlipidemic effects of ethanolic extract of leaves of *Punica granatum* in alloxan-induced non-insulin-dependent diabetes mellitus albino rats. Indian J Pharmacol.

[CR39] Davidson MH, Maki KC, Dicklin MR (2009). Effects of consumption of pomegranate juice on carotid intima-media thickness in men and women at moderate risk for coronary heart disease. Am J Cardiol.

[CR40] de O. Silva L, Ranquine LG, Monteiro M, Torres AG, (2019). Pomegranate (*Punica granatum* L.) seed oil enriched with conjugated linolenic acid (cLnA), phenolic compounds and tocopherols: improved extraction of a specialty oil by supercritical CO2. J Supercrit Fluids.

[CR01] De Hoya M, Mata P (1989) Estuido de los distintos components de la dietary la aterosclerosis. In: Commission EC (ed) Boletín Campaña difusión conocimiento científico sobre el Aceite de Oliva. Unión Europea, Madrid, pp 1–4

[CR41] Deng Y, Li Y, Yang F (2017). The extract from Punica granatum (pomegranate) peel induces apoptosis and impairs metastasis in prostate cancer cells. Biomed Pharmacother.

[CR42] Derakhshan Z, Ferrante M, Tadi M (2018). Antioxidant activity and total phenolic content of ethanolic extract of pomegranate peels, juice and seeds. Food Chem Toxicol.

[CR43] Di Stefano V, Pitonzo R, Novara ME (2019). Antioxidant activity and phenolic composition in pomegranate (*Punica granatum* L.) genotypes from south Italy by UHPLC-Orbitrap-MS approach. J Sci Food Agric.

[CR44] Dludla P, Nkambule B, Jack B (2018). Inflammation and oxidative stress in an obese state and the protective effects of gallic acid. Nutrients.

[CR45] DuLi LJ, Zhan X (2019). Pomegranate peel polyphenols inhibits inflammation in LPS-induced RAW2647 macrophages via the suppression of TLR4/NF-κB pathway activation. Food Nutr Res.

[CR46] Durgawale P, Datkhile K (2016) Study of In-vitro Anti-Cancer and Anti-Oxidative Properties of Aqueous Extract of Punica Granatum Flowers Nanotechnology Project View project Breast Cancer and Genetic Polymorphisms View project

[CR47] El KC, Ferchichi A, Attia F, Bouajila J (2011). Pomegranate (*Punica granatum*) juices: chemical composition, micronutrient cations, and antioxidant capacity. J Food Sci.

[CR48] Elbatanony MM, El-Feky AM, Hemdan BA, Azab El-Liethy M (2019). Assessment of the antimicrobial activity of the lipoidal and pigment extracts of *Punica granatum* L. leaves. Acta Ecol Sin.

[CR49] Elfalleh W, Hannachi H, Tlili N (2012). Total phenolic contents and antioxidant activities of pomegranate peel, seed, leaf and flower. J Med Plants Res.

[CR50] El-Sheshtawy RI, El-Sisy GA, El-Nattat WS (2016). Effects of pomegranate juice in tris-based extender on cattle semen quality after chilling and cryopreservation. Asian Pacific J Reprod.

[CR51] Fawole OA, Makunga NP, Opara UL (2012). Antibacterial, antioxidant and tyrosinase-inhibition activities of pomegranate fruit peel methanolic extract. BMC Complement Altern Med.

[CR52] Fazeli MR, Bahmani S, Jamalifar H, Samadi N (2011). Effect of probiotication on antioxidant and antibacterial activities of pomegranate juices from sour and sweet cultivars. Nat Prod Res.

[CR53] Fernandes L, Pereira JA, Lopéz-Cortés I (2017). Physicochemical composition and antioxidant activity of several pomegranate (*Punica granatum* L.) cultivars grown in Spain. Eur Food Res Technol.

[CR54] Fischer UA, Carle R, Kammerer DR (2011). Identification and quantification of phenolic compounds from pomegranate (*Punica granatum* L.) peel, mesocarp, aril and differently produced juices by HPLC-DAD–ESI/MSn. Food Chem.

[CR55] Fuster-Muñoz E, Roche E, Funes L (2016). Effects of pomegranate juice in circulating parameters, cytokines, and oxidative stress markers in endurance-based athletes: a randomized controlled trial. Nutrition.

[CR56] Gabizon R, Ovadia H, Abramsky O, et al (2019) Pomegranate oil for preventing and treating neurodegenerative diseases

[CR57] García-Villalba R, Espín JC, Aaby K (2015). Validated method for the characterization and quantification of extractable and nonextractable ellagitannins after acid hydrolysis in pomegranate fruits, juices, and extracts. J Agric Food Chem.

[CR58] García-Villalba R, Giménez-Bastida JA, Ávila-Gálvez MA (2020). Ellagitannins and their gut microbiota-derived metabolites: urolithins.

[CR59] Gbinigie OA, Onakpoya IJ, Spencer EA (2017). Evidence for the effectiveness of pomegranate supplementation for blood pressure management is weak: a systematic review of randomized clinical trials. Nutr Res.

[CR60] Ghavipour M, Sotoudeh G, Tavakoli E (2017). Pomegranate extract alleviates disease activity and some blood biomarkers of inflammation and oxidative stress in Rheumatoid Arthritis patients. Eur J Clin Nutr.

[CR61] Giamogante F, Marrocco I, Cervoni L (2018). Punicalagin, an active pomegranate component, is a new inhibitor of PDIA3 reductase activity. Biochimie.

[CR62] Gil M, Tomá S-Barberán F, Hess-Pierce B, (2000). Antioxidant activity of pomegranate juice and its relationship with phenolic composition and processing. J Agric Food Chem.

[CR02] Gilg E, Schürhoff P (1959) Curso de botánica general y aplicada. Labor S.A, Barcelona

[CR63] Glazer I, Masaphy S, Holland D (2012). Partial identification of antifungal compounds from *Punica granatum* peel extracts. J Agric Food Chem.

[CR64] González-Ortiz M, Martínez-Abundis E, Espinel-Bermúdez MC, Pérez-Rubio KG (2011). Effect of pomegranate juice on insulin secretion and sensitivity in patients with obesity. Ann Nutr Metab.

[CR65] González-Sarrías A, García-Villalba R, Núñez-Sánchez MÁ (2015). Identifying the limits for ellagic acid bioavailability: a crossover pharmacokinetic study in healthy volunteers after consumption of pomegranate extracts. J Funct Foods.

[CR66] González-Trujano ME, Pellicer F, Mena P (2015). Antinociceptive and anti-inflammatory activities of a pomegranate (*Punica granatum* L.) extract rich in ellagitannins. Int J Food Sci Nutr.

[CR67] Govindappa M (2015). A review on role of plant(s) extracts and its phytochemicals for the management of diabetes. J Diabetes Metab.

[CR03] Grande F (1988) El papel de las lipoproteinas de alta densidad (HDL). In: Commission EC (ed) Boletin Europeo. Madrid, pp 2–4

[CR68] Gullon B, Pintado ME, Pérez-Álvarez JA, Viuda-Martos M (2016). Assessment of polyphenolic profile and antibacterial activity of pomegranate peel (*Punica granatum*) flour obtained from co-product of juice extraction. Food Control.

[CR69] Gupta P, Choudhury S, Ghosh S (2019). Dietary pomegranate supplement alleviates murine pancreatitis by modulating Nrf2-p21 interaction and controlling apoptosis to survival switch. J Nutr Biochem.

[CR70] Haghayeghi K, Shetty K, Labbé R (2013). Inhibition of foodborne pathogens by pomegranate juice. J Med Food.

[CR71] Hanani ZAN, Yee FC, Nor-Khaizura MAR (2019). Effect of pomegranate (*Punica granatum* L.) peel powder on the antioxidant and antimicrobial properties of fish gelatin films as active packaging. Food Hydrocoll.

[CR04] Harborne J (1982) The Flavonoids, 1st edn. Springer US

[CR72] Hartman RE, Shah A, Fagan AM (2006). Pomegranate juice decreases amyloid load and improves behavior in a mouse model of Alzheimer’s disease. Neurobiol Dis.

[CR73] Hayrapetyan H, Hazeleger WC, Beumer RR (2012). Inhibition of Listeria monocytogenes by pomegranate (*Punica granatum*) peel extract in meat paté at different temperatures. Food Control.

[CR74] Heber D, Seeram NP, Wyatt H (2007). Safety and antioxidant activity of a pomegranate ellagitannin-enriched polyphenol dietary supplement in overweight individuals with increased waist size. J Agric Food Chem.

[CR75] Heena J, Ashraf Pal M, Hamdani H (2018). Antioxidant activity of pomegranate peel and seed powder extracts. J Pharmacogn Phytochem.

[CR76] Hmid I, Elothmani D, Hanine H (2017). Comparative study of phenolic compounds and their antioxidant attributes of eighteen pomegranate (*Punica granatum* L.) cultivars grown in Morocco. Arab J Chem.

[CR77] Hmid I, Hanine H, Elothmani D, Oukabli A (2018). The physico-chemical characteristics of Morrocan pomegranate and evaluation of the antioxidant activity for their juices. J Saudi Soc Agric Sci.

[CR78] Holland D, Bar-Ya’akov I (2018) Pomegranate (*Punica Granatum* L.) Breeding. Advances in Plant Breeding Strategies: fruits. Springer International Publishing, Geramany. pp 601–647

[CR79] Hosseini B, Saedisomeolia A, Wood LG (2016). Effects of pomegranate extract supplementation on inflammation in overweight and obese individuals: a randomized controlled clinical trial. Complement Ther Clin Pract.

[CR80] Husain H, Latief U, Ahmad R (2018). Pomegranate action in curbing the incidence of liver injury triggered by Diethylnitrosamine by declining oxidative stress via Nrf2 and NFκB regulation. Sci Rep.

[CR81] International Food Information Service (2009) Dictionary of food science and technology, 2nd edn. Wiley-Blackwell

[CR82] IVIA (2017) Obtainment of new pomegranate varieties program

[CR83] Janardan Salwe K, Salwe KJ, Sachdev D (2014). Evaluation of antinociceptive and anti-inflammatory effect of the hydroalcoholic extracts of leaves and fruit peel of *P. Granatum* in experimental animals. Asian J Pharm Clin Res.

[CR84] Jasuja N, Saxena R, Chandra S, Sharma R (2012). Pharmacological characterization and beneficial uses of *Punica granatum*. Asian J Plant Sci.

[CR05] Jayakumar S, Madankumar A, Asokkumar S (2012). Potential preventive effect of carvacrol against diethylnitrosamine-induced hepatocellularcarcinoma in rats. Mol Cell Biochem.

[CR85] Juneja VK, Cadavez V, Gonzales-Barron U (2016). Effect of pomegranate powder on the heat inactivation of *Escherichia coli* O104:H4 in ground chicken. Food Control.

[CR86] Kamali M, Tavakoli H, Khodadoost M (2015). Efficacy of the *Punica granatum* peels aqueous extract for symptom management in ulcerative colitis patients. A randomized, placebo-controlled, clinical trial. Complement Ther Clin Pract.

[CR87] Kaplan M, Hayek T, Raz A (2001). Pomegranate juice supplementation to atherosclerotic mice reduces macrophage lipid peroxidation, cellular cholesterol accumulation and development of atherosclerosis. J Nutr.

[CR88] Karimi M, Sadeghi R, Kokini J (2017). Pomegranate as a promising opportunity in medicine and nanotechnology. Trends Food Sci Technol.

[CR89] Kasimsetty SG, Bialonska D, Reddy MK (2010). Colon cancer chemopreventive activities of pomegranate ellagitannins and urolithins. J Agric Food Chem.

[CR90] Kerimi A, Nyambe-Silavwe H, Gauer JS (2017). Pomegranate juice, but not an extract, confers a lower glycemic response on a high–glycemic index food: randomized, crossover, controlled trials in healthy subjects. Am J Clin Nutr.

[CR91] Khadivi A, Ayenehkar D, Kazemi M, Khaleghi A (2018). Phenotypic and pomological characterization of a pomegranate (*Punica granatum *L.) germplasm collection and identification of the promising selections. Sci Hortic (Amsterdam).

[CR92] Khalil AA, Khan MR, Shabbir MA, Rahman KU (2017). Comparison of antioxidative potential and punicalagin content of pomegranate peels. J Animimal Plant Sci.

[CR93] Kharchoufi S, Licciardello F, Siracusa L (2018). Antimicrobial and antioxidant features of ‘Gabsiʼ pomegranate peel extracts. Ind Crops Prod.

[CR94] Kharchoufi S, Parafati L, Licciardello F (2018). Edible coatings incorporating pomegranate peel extract and biocontrol yeast to reduce *Penicillium digitatum* postharvest decay of oranges. Food Microbiol.

[CR95] Khateeb J, Gantman A, Kreitenberg AJ (2010). Paraoxonase 1 (PON1) expression in hepatocytes is upregulated by pomegranate polyphenols: a role for PPAR-γ pathway. Atherosclerosis.

[CR96] Khwairakpam AD, Bordoloi D, Thakur KK (2018). Possible use of *Punica granatum* (Pomegranate) in cancer therapy. Pharmacol Res.

[CR97] Kim ND, Mehta R, Yu W (2002). Chemopreventive and adjuvant therapeutic potential of pomegranate (*Punica granatum*) for human breast cancer. Breast Cancer Res Treat.

[CR98] Kiraz Y, Neergheen-Bhujun VS, Rummun N, Baran Y (2016). Apoptotic effects of non-edible parts of *Punica granatum* on human multiple myeloma cells. Tumor Biol.

[CR99] Kujawska M, Jourdes M, Kurpik M (2019). Neuroprotective effects of pomegranate juice against parkinson’s disease and presence of ellagitannins-derived metabolite—urolithin a—in the brain. Int J Mol Sci.

[CR100] Kumar N, Neeraj D (2018). Study on physico-chemical and antioxidant properties of pomegranate peel. J Pharmacogn Phytochem.

[CR101] Kumar D, Singh S, Singh AK, Rizvi SI (2013). Pomegranate (*Punica granatum*) peel extract provides protection against mercuric chloride-induced oxidative stress in Wistar strain rats. Pharm Biol.

[CR102] Kutan Fenercioglu A, Saler T, Genc E (2010). The effects of polyphenol-containing antioxidants on oxidative stress and lipid peroxidation in type 2 diabetes mellitus without complications. J Endocrinol Invest.

[CR103] Lafay S, Gil-Izquierdo A (2008). Bioavailability of phenolic acids Phytochem Rev.

[CR104] Lansky EP, Newman RA (2007). Punica granatum (pomegranate) and its potential for prevention and treatment of inflammation and cancer. J Ethnopharmacol.

[CR105] Lansky EP, Jiang W, Mo H (2005). Possible synergistic prostate cancer suppression by anatomically discrete pomegranate fractions. Invest New Drugs.

[CR106] Larrosa M, González-Sarrías A, Yáñez-Gascón MJ (2010). Anti-inflammatory properties of a pomegranate extract and its metabolite urolithin-A in a colitis rat model and the effect of colon inflammation on phenolic metabolism. J Nutr Biochem.

[CR107] Lepionka T, Białek A, Białek M (2019). Mammary cancer risk and serum lipid profile of rats supplemented with pomegranate seed oil and bitter melon extract. Prostaglandins Other Lipid Mediat.

[CR108] Les F, Arbonés-Mainar JM, Valero MS, López V (2018). Pomegranate polyphenols and urolithin A inhibit α-glucosidase, dipeptidyl peptidase-4, lipase, triglyceride accumulation and adipogenesis related genes in 3T3-L1 adipocyte-like cells. J Ethnopharmacol.

[CR109] Li X, Wasila H, Liu L (2015). Physicochemical characteristics, polyphenol compositions and antioxidant potential of pomegranate juices from 10 Chinese cultivars and the environmental factors analysis. Food Chem.

[CR110] Li Y, Yang F, Zheng W (2016). *Punica granatum* (pomegranate) leaves extract induces apoptosis through mitochondrial intrinsic pathway and inhibits migration and invasion in non-small cell lung cancer in vitro. Biomed Pharmacother.

[CR111] Liu W, Ma H, Frost L (2014). Pomegranate phenolics inhibit formation of advanced glycation endproducts by scavenging reactive carbonyl species. Food Funct.

[CR112] Lopez-Lazaro M (2009). Distribution and biological activities of the flavonoid luteolin. Mini-Reviews Med Chem.

[CR113] Lucci P, Pacetti D, Loizzo MR, Frega NG (2015). *Punica granatum* cv. Dente di Cavallo seed ethanolic extract: antioxidant and antiproliferative activities. Food Chem.

[CR114] Makino-Wakagi Y, Yoshimura Y, Uzawa Y (2012). Ellagic acid in pomegranate suppresses resistin secretion by a novel regulatory mechanism involving the degradation of intracellular resistin protein in adipocytes. Biochem Biophys Res Commun.

[CR115] Malviya S, Alok J, Hettiarachchy N (2014). Antioxidant and antibacterial potential of pomegranate peel extracts. J Food Sci Technol.

[CR116] Mandal A, Bhatia D, Bishayee A (2017). Anti-inflammatory mechanism involved in pomegranate-mediated prevention of breast cancer: the role of NF-κB and Nrf2 signaling pathways. Nutrients.

[CR117] Mansour E, Ben KA, Lachiheb B (2013). Phenolic compounds, antioxidant, and antibacterial activities of peel extract from tunisian pomegranate. J Agron Sci Technol.

[CR118] Mansoury M (2019). Evidence-based therapeutic activity of pomegranate and its active constituent ellagic acid. Pharmacophore.

[CR119] MAPA (2019) Informe Del Consumo Alimentario En España 2018. Madrid

[CR120] Martínez L, Castillo J, Ros G (2019). Antioxidant and antimicrobial activity of rosemary, pomegranate and olive extracts in fish patties. Antioxidants.

[CR121] Mastrogiovanni F, Mukhopadhya A, Lacetera N (2019). Anti-inflammatory effects of pomegranate peel extracts on in vitro human intestinal Caco-2 cells and ex vivo porcine colonic tissue explants. Nutrients.

[CR122] Matthaiou CM, Goutzourelas N, Stagos D (2014). Pomegranate juice consumption increases GSH levels and reduces lipid and protein oxidation in human blood. Food Chem Toxicol.

[CR123] Mazumder MK, Choudhury S, Borah A (2019). An in silico investigation on the inhibitory potential of the constituents of pomegranate juice on antioxidant defense mechanism: relevance to neurodegenerative diseases. IBRO Reports.

[CR07] Melgarejo P (1993) Selección y tipificación varietal de granado (Punica granatum L.). Universidad Politécnica de Valencia

[CR09] Melgarejo P, Salazar D (2003) Tratado de fruticultura para zonas áridas y semiáridas, Vol. II: Algarrobo, granado y jinjolero. AMV Ediciones, Madrid

[CR08] Melgarejo P, Calín-Sánchez Á, Carbonell-Barrachina ÁA (2015). Antioxidant activity, volatile composition and sensory profile of four new very-early apricots (Prunus armeniaca L.). J Sci Food Agric.

[CR124] Melgarejo-Sánchez P, Martínez JJ, Hernández F et al (2015) The pomegranate tree in the world: new cultivars and uses. Acta Hortic 1089 327–332. 10.17660/ActaHortic.2015.1089.43

[CR125] Mirmiran P, Fazeli MR, Asghari G (2010). Effect of pomegranate seed oil on hyperlipidaemic subjects: a double-blind placebo-controlled clinical trial. Br J Nutr.

[CR126] Mithun S, Sreedam Chandra D, Sajal Kumar S (2012). Analgesic and anti-inflammatory activities of flower extracts of *Punica granatum* Linn. (Punicaceae). J Appl Pharm Sci.

[CR127] Mohamad Sukri SNA, Shameli K, Mei-Theng Wong M (2019). Cytotoxicity and antibacterial activities of plant-mediated synthesized zinc oxide (ZnO) nanoparticles using Punica granatum (pomegranate) fruit peels extract. J Mol Struct.

[CR128] Moher D, Shamseer L, Clarke M (2015). Preferred reporting items for systematic review and meta-analysis protocols (PRISMA-P) 2015 statement. Syst Rev.

[CR129] Morzelle MC, Salgado JM, Telles M (2016). Neuroprotective effects of pomegranate peel extract after chronic infusion with amyloid-β peptide in mice. PLoS ONE.

[CR130] Mphahlele RR, Fawole OA, Makunga NP, Linus Opara U (2017). Functional properties of pomegranate fruit parts: influence of packaging systems and storage time. J Food Meas Charact.

[CR131] Nasiri E, Hosseinimehr SJ, Akbari J (2017). The effects of *Punica granatum* flower extract on skin injuries induced by burn in rats. Adv Pharmacol Sci.

[CR132] Naziri Z, Rajaian H, Firouzi R (2012). Antibacterial effects of Iranian native sour and sweet pomegranate (*Punica granatum*) peel extracts against various pathogenic bacteria. Iran J Vet Res..

[CR133] Nekooeian AA, Eftekhari MH, Adibi S, Rajaeifard A (2014). Effects of pomegranate seed oil on insulin release in rats with type 2 diabetes. Iran J Med Sci.

[CR134] Nirwana I (2018). Application of pomegranate (*Punica granatum* Linn.) fruit extract for accelerating post-tooth extraction wound healing. Dent J (Majalah Kedokt Gigi).

[CR135] Olapour S, Najafzadeh H (2010). Evaluation analgesic, anti-inflammatory and antiepileptic effect of hydro alcoholic peel extract of “*Punica granatum* (pomegranate)”. Asian J Med Sci.

[CR136] Opara LU, Al-Ani MR, Al-Shuaibi YS (2009). Physico-chemical properties, vitamin c content, and antimicrobial properties of pomegranate fruit (*Punica granatum* L.). Food Bioprocess Technol.

[CR137] Orak HH, Yagar H, Isbilir SS (2012). Comparison of antioxidant activities of juice, peel, and seed of pomegranate (*Punica granatum* L.) and inter-relationships with total phenolic, Tannin, anthocyanin, and flavonoid contents. Food Sci Biotechnol.

[CR138] Ouachrif A, Khalki H, Chaib S (2012). Comparative study of the anti-inflammatory and antinociceptive effects of two varieties of *Punica granatum*. Pharm Biol.

[CR139] Pagliarulo C, De Vito V, Picariello G (2016). Inhibitory effect of pomegranate (*Punica granatum* L.) polyphenol extracts on the bacterial growth and survival of clinical isolates of pathogenic Staphylococcus aureus and *Escherichia coli*. Food Chem.

[CR140] Panichayupakaranant P, Tewtrakul S, Yuenyongsawad S (2010). Antibacterial, anti-inflammatory and anti-allergic activities of standardised pomegranate rind extract. Food Chem.

[CR141] Pantuck AJ, Leppert JT, Zomorodian N (2006). Phase II study of pomegranate juice for men with rising prostate-specific antigen following surgery or radiation for prostate cancer. Clin Cancer Res.

[CR142] Poyrazoğlu E, Gökmen V, Artιk N (2002). Organic acids and phenolic compounds in pomegranates (*Punica granatum* L.) grown in Turkey. J Food Compos Anal.

[CR143] Prithviraj K (2018). BIological activities of flavonoids: an overview. Int J Pharm Sci Res.

[CR144] Rocha A, Wang L, Penichet M, Martins-Green M (2012). Pomegranate juice and specific components inhibit cell and molecular processes critical for metastasis of breast cancer. Breast Cancer Res Treat.

[CR145] Rojanathammanee L, Puig KL, Combs CK (2013). Pomegranate polyphenols and extract inhibit nuclear factor of activated T-Cell activity and microglial activation in vitro and in a transgenic mouse model of alzheimer disease. J Nutr.

[CR146] Rosas-Burgos EC, Burgos-Hernández A, Noguera-Artiaga L (2017). Antimicrobial activity of pomegranate peel extracts as affected by cultivar. J Sci Food Agric.

[CR147] Rosenblat M, Hayek T, Aviram M (2006). Anti-oxidative effects of pomegranate juice (PJ) consumption by diabetic patients on serum and on macrophages. Atherosclerosis.

[CR148] Sahebkar A, Gurban C, Serban A (2016). Effects of supplementation with pomegranate juice on plasma C-reactive protein concentrations: a systematic review and meta-analysis of randomized controlled trials. Phytomedicine.

[CR149] Sarma P, Das S (2014). A study on the anticonvulsant and antianxiety activity of ethanolic extract of *Punica granatum* Linn. Int J Pharm Pharm Sci..

[CR150] Šavikin K, Živković J, Alimpić A (2018). Activity guided fractionation of pomegranate extract and its antioxidant, antidiabetic and antineurodegenerative properties. Ind Crops Prod.

[CR151] Seeram NP, Lee R, Heber D (2004). Bioavailability of ellagic acid in human plasma after consumption of ellagitannins from pomegranate (*Punica granatum* L.) juice. Clin Chim Acta.

[CR152] Seeram NP, Adams LS, Henning SM (2005). In vitro antiproliferative, apoptotic and antioxidant activities of punicalagin, ellagic acid and a total pomegranate tannin extract are enhanced in combination with other polyphenols as found in pomegranate juice. J Nutr Biochem.

[CR153] Seeram NP, Aronson WJ, Zhang Y (2007). Pomegranate ellagitannin-derived metabolites inhibit prostate cancer growth and localize to the mouse prostate gland. J Agric Food Chem.

[CR010] Seeram NP, Zhang Y, McKeever R (2008). Pomegranate juice and extracts provide similar levels of plasma and urinary ellagitannin metabolites in human subjects. J Med Food.

[CR154] Setiadhi R, Sufiawati I (2017) Fractionation of Red Pomegranate (Punica granatum L.) Seed Ethanolic Extracts for Identifying Active Compounds. In: - The 7th International Meeting and The 4th Joint Scientific Meeting in Dentistry. Shangri, pp 277–280

[CR155] Shahrzad S, Bitsch I (1998). Determination of gallic acid and its metabolites in human plasma and urine by high-performance liquid chromatography. J Chromatogr B Biomed Sci Appl.

[CR156] Shahrzad S, Aoyagi K, Winter A (2001). Pharmacokinetics of gallic acid and its relative bioavailability from tea in healthy humans. J Nutr.

[CR157] Shirode AB, Kovvuru P, Chittur SV (2014). Antiproliferative effects of pomegranate extract in MCF-7 breast cancer cells are associated with reduced DNA repair gene expression and induction of double strand breaks. Mol Carcinog.

[CR158] Shirode AB, Bharali DJ, Nallanthighal S (2015). Nanoencapsulation of pomegranate bioactive compounds for breast cancer chemoprevention. Int J Nanomedicine.

[CR159] Siddarth P, Li Z, Miller KJ (2020). Randomized placebo-controlled study of the memory effects of pomegranate juice in middle-aged and older adults. Am J Clin Nutr.

[CR160] Sidhu JS, Zafar TA (2012). Super Fruits: Pomegranate, Wolfberry, Aronia (Chokeberry), Acai, Noni, and Amla. Handbook of Fruits and Fruit Processing.

[CR161] Sohrab G, Roshan H, Ebrahimof S (2019). Effects of pomegranate juice consumption on blood pressure and lipid profile in patients with type 2 diabetes: a single-blind randomized clinical trial. Clin Nutr ESPEN.

[CR162] Still DW, Seeram N, Schulman R, Heber D (2006). Pomegranates: a botanical perspective. Pomegranates: ancient roots to modern medicine.

[CR163] Subash S, Essa MM, Al-Asmi A (2014). Pomegranate from oman alleviates the brain oxidative damage in transgenic mouse model of alzheimer’s disease. J Tradit Complement Med.

[CR164] Subash S, Braidy N, Essa MM (2015). Long-term (15 mo) dietary supplementation with pomegranates from Oman attenuates cognitive and behavioral deficits in a transgenic mice model of Alzheimer’s disease. Nutrition.

[CR165] Sudheesh S, Soumya K, Jesna J (2018). A novel chalcone derivative from *Punica granatum* peel inhibits LOX/COX enzyme activity. Beni-Suef Univ J Basic Appl Sci.

[CR166] Suman M, Bhatnagar P (2019). A review on proactive pomegranate one of the healthiest foods. Int J Chem Stud.

[CR167] Sumner MD, Elliott-Eller M, Weidner G (2005). Effects of pomegranate juice consumption on myocardial perfusion in patients with coronary heart disease. Am J Cardiol.

[CR168] Syeda S, Kumar S, Pushpalatha C, Mohsin M (2018). Experimental evaluation of antiinflammatory activity of *Punica granatum* peel extract in albino rats. J Chalmeda Anand Rao Inst Med Sci.

[CR169] Tamamm AM, El-Sonbaty SM, Moawed FS, Kandil EI (2018). Antitumor efficacy of ellagic acid against MCF-7 using nanotechnology. Nat Sci.

[CR170] Tehranifar A, Selahvarzi Y, Kharrazi M, Bakhsh VJ (2011). High potential of agro-industrial by-products of pomegranate (*Punica granatum* L.) as the powerful antifungal and antioxidant substances. Ind Crops Prod.

[CR171] Tomás-Barberán FA, González-Sarrías A, García-Villalba R, et al (2017) Urolithins, the rescue of “old” metabolites to understand a “new” concept: Metabotypes as a nexus among phenolic metabolism, microbiota dysbiosis, and host health status. Mol. Nutr. Food Res. 61 10.1002/mnfr.201500901 27158799

[CR172] Tsuzuki T, Kawakami Y, Abe R (2006). Conjugated linolenic acid is slowly absorbed in rat intestine, but quickly converted to conjugated linoleic acid. J Nutr.

[CR173] Vaithiyanathan S, Naveena BM, Muthukumar M (2011). Effect of dipping in pomegranate (*Punica granatum*) fruit juice phenolic solution on the shelf life of chicken meat under refrigerated storage (4 °C). Meat Sci.

[CR174] Velagapudi R, Baco G, Khela S (2016). Pomegranate inhibits neuroinflammation and amyloidogenesis in IL-1β-stimulated SK-N-SH cells. Eur J Nutr.

[CR175] Verma N, Mohanty A, Lal A (2010). Pomegranate genetic resources and germplasm conservation: a review. Fruit, Veg Cereal Sci Biotechnol.

[CR176] Verotta L, Panzella L, Antenucci S (2018). Fermented pomegranate wastes as sustainable source of ellagic acid: antioxidant properties, anti-inflammatory action, and controlled release under simulated digestion conditions. Food Chem.

[CR177] Vroegrijk IOCM, van Diepen JA, van den Berg S (2011). Pomegranate seed oil, a rich source of punicic acid, prevents diet-induced obesity and insulin resistance in mice. Food Chem Toxicol.

[CR011] Viuda-Martos M, Fernández-López J, Pérez-Álvarez JA (2010). Pomegranate and its many functional components as related to human health: a review. Compr Rev Food Sci Food Saf.

[CR178] Wafa BA, Makni M, Ammar S (2017). Antimicrobial effect of the Tunisian Nana variety *Punica granatum *L. extracts against Salmonella enterica (serovars Kentucky and Enteritidis) isolated from chicken meat and phenolic composition of its peel extract. Int J Food Microbiol.

[CR179] Wang L, Ho J, Glackin C, Martins-Green M (2012) Specific Pomegranate Juice Components as Potential Inhibitors of Prostate Cancer Metastasis. Transl Oncol 5:344-IN5. 10.1593/TLO.12190 10.1593/tlo.12190 PMC3470115 23066443

[CR180] Wang B-S, Leu K-L, Huang G-J (2014). Protective effects of an aqueous *Pericarpium Granati* extract against inflammatory damage in mice. J Funct Foods.

[CR181] Watson R, Preedy V (2013). Bioactive food as dietary interventions for liver and gastrointestinal disease.

[CR182] Yuan G-F, Wahlqvist ML, Yuan J-Q (2009). Effect of punicic acid naturally occurring in food on lipid peroxidation in healthy young humans. J Sci Food Agric.

[CR183] Yuan T, Ma H, Liu W (2016). Pomegranate’s neuroprotective effects against Alzheimer’s disease are mediated by urolithins, its ellagitannin-gut microbial derived metabolites. ACS Chem Neurosci.

[CR184] Yuniarti WM, Primarizky H, Lukiswanto BS (2018). The activity of pomegranate extract standardized 40% ellagic acid during the healing process of incision wounds in albino rats (Rattus norvegicus). Vet Word.

[CR185] Zarei A (2017). Biochemical and pomological characterizaion of pomegranate accessions in Fars province of Iran. SABRAO J Breed Genet.

[CR186] Zehra T, Ahmed S, Zehra S (2019). Review of characteristic components, traditional and pharmacological properties of *Punica granatum*. RADS J Pharm Pharm Sci.

[CR187] Zhang L, Gao Y, Zhang Y (2010). Changes in bioactive compounds and antioxidant activities in pomegranate leaves. Sci Hortic (Amsterdam).

[CR188] Zhou P, Li L-P, Luo S-Q (2007). Intestinal absorption of luteolin from peanut hull extract is more efficient than that from individual pure luteolin. J Agric Food Chem.

[CR189] Zuriaga E, Bartual J, Pintová J, Badenes ML (2017) Genetic diversity among pomegranate germplasm assessed by microsatellite markers. In: IV International Symposium on Pomegranate and Minor Mediterranean Fruits. International Society for Horticultural Science, Elche, p 19

